# Supplementary material for: Critical calls: Circadian and seasonal periodicity in vocal activity in a breeding colony of Panamanian golden frogs (Atelopus zeteki)
Source: PLoS One. 2023 Aug 17;18(8):e0286582. doi: 10.1371/journal.pone.0286582 (PMC10434951; doi:10.1371/journal.pone.0286582)
Supplement: Attachment — Submitted filename: Response to Reviewers.docx [file pone.0286582.s001.docx]

May 11, 2023

Dear Dr. Cooper,

We greatly appreciate the constructive and critical feedback from the two reviewers. Please find our responses below to each of the reviewer’s comments. Along with this letter, you will also find in our submission the revised manuscript with and without tracked changes as well as revised images of our figures. We believe that we have both appropriately formatted the manuscript to adhere to PLOS One’s style requirements and responded to all reviewer comments adequately, but of course we will gladly revisit and revise any components if need be. We look forward to hearing back from you.

Sincerely,

Alan Zigler, B.S.

The Maryland Zoo in Baltimore

Tel. 215 384 6811

Alan.Zigler@MarylandZoo.org

**Reviewer 1**

1. First, please explain in more detail your individual call recording procedure.  Group housing conditions are well described, but how individuals were transferred and housed individually is not sufficiently explained.  Please see corresponding concern from the Reviewer regarding this issue.  Include a complete description of the process required for individual housing (time given to acclimate to the new cage), dimensions, etc.  Further, a description of how individual housing vs. social housing might affect vocal behavior would be relevant - note Reviewer comment on this as well.

RESPONSE: We have added detail explaining individual housing and acoustic monitoring in the method section.

2. Second, I have one concern about statistical analyses, and this centers on your bivariate correlation analyses of body mass features and acoustic features.  First, this analysis is not described in the methods.  Second, your analysis is challenging due to the physical differences between the two morphotypes, that have non-overlapping groups of data - I feel the data would be stronger if your slopes for the regression correlations within each morphotype were not significantly different from each other, however you lack sufficient statistical power to test this idea (in my estimation based on the sample sizes for each morphotype).  I would like to suggest collecting more data for this analysis, but I am sympathetic to the challenges of this experiment.  I suggest you consider alternative analysis options or give caveats for this section due to these group differences. Last issue here, when reporting a simple regression, a lower case "r" should be used.  The upper case R indicates multiple regression.

RESPONSE: Unfortunately, it is not possible to add more individuals to the analysis. Therefore we have revised the discussion to explain the limitations of the current analysis, and we have reduced/condensed the discussion to not over-state the relevance of the correlational results. The methods were revised to include information on the Pearson correlation analysis.

3. Third, and last, in my view, Figure 1 would more clearly show spectral content differences in the calls if the power spectrum was displayed to the left side of the spectrogram.  This would help to clarify what you mean by the first and second dominant frequencies.  In the methods, please briefly explain how the first and second dominant frequency are determined. Please note Reviewer concerns regarding this issue as well.

RESPONSE: Figure 1 has been revised as suggested.

**Reviewer 2**

1. Line 185 – When investigating the population’s vocal activity how you count the total number of calls hourly. The recordings might have lot of overlapping calls and many low amplitude calls. Normally toads are making choruses.

RESPONSE: Our process for counting calls within a day’s recordings allowed us the opportunity to catch any overlapping calls – we would see patterned single calls within a generated spectrogram and play back audio for any recordings that do not look to fit a standard call structure, which we could confidently use to confirm if the recording included overlap. To miss low amplitude calls is a valid concern, however, the vocal activity measure used here is likely to be a representation of overall population-wide activity because toads tend to make choruses. Furthermore, we took great care to confirm that frog calls originating in any tank would trigger recording. Observations during the initial month-long setup we confirmed by simultaneous observations of tanks with calling toads and the AVISOFT recorder that sensitivity was sufficient to capture calls from each tank. We have added additional detail to this section of the methodology to address this.

2. Line 227– In introduction section good to have a separate paragraph to explain the physiological aspects of calling such as metabolic rate, hormonal state, etc….

RESPONSE: We appreciate the suggestion by the reviewer. Please note that we indicate in the first paragraph of the introduction that “costs for calling are estimated to be 10 to 25 times greater than the resting metabolic rate.” Furthermore the second paragraph highlights the importance of the hormonal state (“Research in diverse anuran species has shown that vocal behavior serves important functions in reproductive biology and provides insight into an animal’s hormonal state and expectations of further reproductive behavior.”). We kindly ask to not further expand the Introduction.

3. Line 223 – How did you select 20 calls from the sample recordings? And they might have periodic differences in call characters.

RESPONSE: We have added additional detail to the method section.

We also acknowledge in the discussion section that future research should increase the individual sample size to explore the effects of season, reproductive state, age, and body size.

4. Line 227– Better explain briefly about the call structure and unique properties of Atelopus zeteki to give a better understanding at the beginning. For example what you mean by first and second dominant frequencies?

RESPONSE: We have revised Figure 1 in order to better explain the acoustic parameters used here.

5. Line 236– Table 1, cant see the complete table

RESPONSE: We have modified the document so that Table 1 is on a separate page in landscape.

6. Line 252– It says CVb/CVw were larger than 1 for the first dominant frequency. But table 2 shows the value is larger than one for all variables. Reword the sentence

RESPONSE: The text has been revised.

7. Line 262– Two individuals from S population means 2/7 = 29%. Increasing sample size might provide much better representation.

RESPONSE: We have revised the discussion and discuss limitations of the current approach and possible routes for future studies.

8. Line 344 – In methodology mentioned the room was acoustically monitored from December 2019 until December 2020? But in figure 6 shown actograms of 2021
RESPONSE: In order to assess the effects of human activity on vocalizations, we needed two additional pairs of comparable 24-hour periods (normal vs. altered schedule) outside of the year we recorded all other calls, which occurred in 2021. We included a paragraph in methodology to better indicate this.

9. I wonder why the calling activities are comparatively higher around 1200 during regular husbandry. Does that indicate the lunch break (absence of the personnel in the frog room)?

RESPONSE: Call activity increases between 1200-1300 when personnel leave the room for a lunch break. This observation is of critical importance and may affect how future husbandry schedules are designed.

10. Line 366 – Everywhere else mentioned the alternative schedule is between 1300 and 1600 ???
RESPONSE: We have corrected this error.

11. Line 374 – First finding is not new. Already known information. Isn’t it?

RESPONSE: We have revised this sentence.

12. Line 433 – Considering the human disturbances it is unfair to claim that they have two peaks. Though the disturbances happen between 1300 to 1600, figure 6B shows almost one cycle in alternative schedule day.

RESPONSE: The periodogram analysis identified two cycles, 24 hours and 12 hours. Additional peaks did not reach threshold. We hope that the revisions of the discussion section sufficiently explain multiple potential sources for disturbing a natural cyclicity. Please note that figure 6B are only three recordings and do not allow conclusions about robust periods in vocal activity.

13. Fig 1– In your figure male 2,3 and 4 clearly show 3-4 frequency bands. You have considered first and second dominant frequency only. And it is not clear which band you selected as second dominant frequency. Further the figure shows they modulate the frequency. So their starting frequency and end frequency is different. Crocroft (1990) has also mentioned about the frequency modulation of the species.

RESPONSE: We have revised Figure 1 to better illustrate the parameters used here.

14. Fig 2 – Draw and show the confidence ellipses.

RESPONSE: Figure 2 has been modified to include confidence ellipses.

15. Good if you could make available some of your call recordings as supplementary materials. Consider deposit in a public repository and share the link.

RESPONSE: Data have been published on DRYAD.

Zigler, Alan; Riede, Tobias (2023), Critical Calls: Circadian and seasonal periodicity in vocal activity in a breeding colony of Panamanian golden frogs (Atelopus zeteki), Dryad, Dataset, <https://doi.org/10.5061/dryad.k98sf7mbx>
